# Anton syndrome after subarachnoid hemorrhage and delayed cerebral ischemia: A case report

**DOI:** 10.1016/j.cccb.2021.100023

**Published:** 2021-06-30

**Authors:** Barojas-Alvarez Manuel Ricardo, Longoria-Ibarrola Erika Mariana, Sosa-Ortiz AL, Calleja-Castillo Juan Manuel, Ramirez-Bermudez Jesus

**Affiliations:** a Neuropsychiatry Unit, National Institute of Neurology and Neurosurgery, Mexico City, Mexico; b Dementia Laboratory, National Institute of Neurology and Neurosurgery, Mexico City, Mexico; c Emergency Department, National Institute of Neurology and Neurosurgery, Mexico City, Mexico

**Keywords:** Anton syndrome, Cotard syndrome, Metacognition, Right hemisphere, Stroke, Subarachnoid hemorrhage

## Abstract

We report the case of a patient with ruptured middle cerebral artery aneurysm, subarachnoid hemorrhage and frontal intracerebral hemorrhage, who secondarily presented with delayed cerebral ischemia and bilateral occipital infarcts, with complete vision loss, visual anosognosia, and confabulations as the main symptoms. Additionally, the patient had defined features of Cotard syndrome, as revealed by persistent nihilistic delusions. The brain imaging studies showed a bilateral occipital ischemic lesion, as well as frontal and parietal hemorrhagic lesions in the right hemisphere. We address the general theories of anosognosia, confabulation and the problem of why a bilateral occipital lesion is not enough for these metacognitive defects to exist. The presence of right frontal and or parietal hemisphere lesions could be necessary features in most cases.

## Highlights

• Bilateral occipital damage is not sufficient for the onset of visual anosognosia.
• Anton syndrome may result from bilateral occipital and right frontoparietal lesions.
• Anosognosia can be understood as a metacognitive deficit.
• Disturbed metacognition is related to right fronto-parietal dysfunction.
• Delusions of being dead (Cotard syndrome) may appear after right hemispheric damage.

## 1 Introduction

Anton syndrome is characterized by visual anosognosia and confabulation associated with cortical blindness [1,2]. Since 1965, less than 30 cases of this syndrome have been published. The reported etiology is variable, including cardio-vascular instability on invasive procedures, adrenoleukodystrophy, and obstetric hemorrhage, among others [3]. An ischemic cerebrovascular event of both posterior cerebral arteries is the most commonly reported cause; and in all the 30 cases described, bilateral occipital cortex lesions have been found [4]. However, it is not clear why most patients with visual loss related to occipital lobe lesions do not develop anosognosia and confabulation. The purpose of this article is to present a case of Anton's syndrome in which visual loss and bilateral occipital damage coexisted with large frontal and parietal lesions in the right hemisphere.

## 2 Case description

JC is a 39-year-old left-handed man with a 5 years history of non-treated systemic arterial hypertension, alcohol, and tobacco use. His-condition began with a biparietal thunderclap headache, and sudden neurological deficit with loss of vision, left-sided weakness, and generalized tonic-clonic seizures. He was evaluated at the National Institute of Neurology and Neurosurgery (NINN) of Mexico. A Sylvian fissure and peri-mesencephalic subarachnoid and frontal and parietal hemorrhages were identified. Angio-tomography study reported a right middle cerebral artery (M1 portion) aneurysm.

Digital subtraction angiography and aneurysm embolization was performed. Despite early treatment, the patient presented vasospasm and delayed cerebral ischemia, resulting in bilateral occipital cerebral hemorrhagic infarctions. Later, ventricular derivation was performed. After surgery, a more severe left leg weakness and hyperactive delirium were reported. All deficits presented improved and stabilized after a few days with specific treatment and rehabilitation. However, JC presented complete visual loss before discharge and he continued his treatment as an outpatient.

During his first week at home, JC tripped over furniture, fell on objects, and had difficulty finding his way around. He stumbled while walking in his house; he would say to his wife: "you have a mess here." JC acted as if he could see and denied the loss of vision. He also said that: “his uncle was dead in his house”, “he was dead", "his house was not his house”. It smelled like "there was something dead in the house". Additionally, he said that there was a dead body in his bed, so he would not sleep there. He would point at supposed scars in his face and say: “I am dead already but still walking, I have been hurt by gunshots.”

On a regular visit, a mental examination was carried out. Despite integrity in alertness, attention, memory, speech, language, mood, affect, he was confabulating, behaving, and speaking as if he had normal vision. A severe impairment of judgment was observed, also he presented nihilistic delusions as described before.

During the second week as an outpatient, there was an increase in motor activity; He would say: "They put things around me to hurt me," and "they want to drive me crazy." According to his family, the patient was unable to see, but "asked for the keys of his yellow truck as it was badly parked." "He wanted to buy beer by driving his vehicle and repeatedly asked for the truck keys." When confronted by his family, he became verbally and physically aggressive.

During a subsequent visit to the hospital, his speed of speech was increased and had a tendency toward verbiage. A pleasant hyper-thymic state of mind was observed. However, he would insist on nihilistic delusions: "I'm already dead," and "they shot me in the body.” The family reported decreased food intake, a lack of adherence to medical treatment, and caregiver fatigue. Hence, he required hospitalization at the Neuropsychiatry Unit of the NINN.

The Neuro-ophthalmology department reported data consistent with Terson syndrome: a severe bilateral vitreous hemorrhage on fundoscopy. During the ophthalmologic exam, JC was found with loss of all visual sensations, including light and dark perception, loss of visual threat reflex, with preservation of photo-motor pupillary reflexes, and of eye movements. The rest of the neurologic examination was normal. Laboratory tests were within normal limits and imaging showed no new brain lesions.

**Fig. 1:**
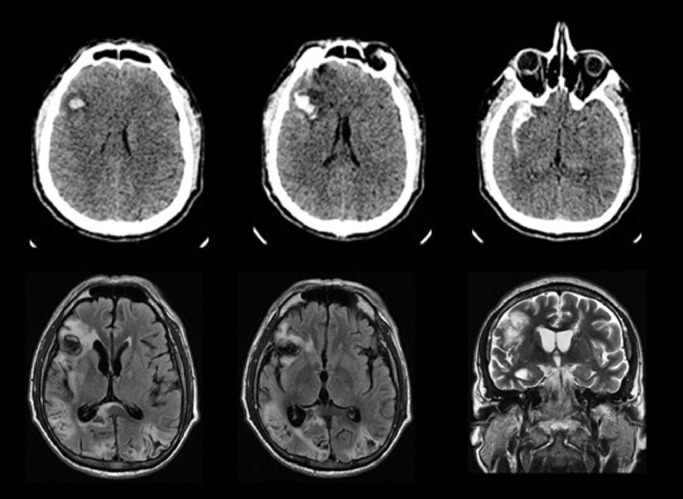
Image studies. Three CT axial sections show a heterogeneous area of ​​intraparenchymal and subarachnoid bleeding, and intracerebral hematoma in the right frontal lobe. Two axial MRI sections in a FLAIR sequence show the extension of the lesion, involving the lateral aspects of the right frontal lobe, with extension to deep white matter, the anterior insular cortex, and the right occipito-temporo-parietal cortex, as well as hemorrhagic infarction in both occipital lobes. A coronal T2 MRI section shows the lesion in the lateral aspect of the right hemisphere

As may be seen in Fig. 1, a CT scan before brain surgery showed a heterogeneous area of intra-parenchymal and subarachnoid bleeding and perilesional edema with a right frontal intra-cerebral hematoma. Two months after the initial hemorrhage, an MRI scan showed an infarction with secondary hemorrhage of the left occipito-parietal and right occipital lobes. Also, a right frontal hemorrhagic lesion was observed.

Two months after the hemorrhage, a neurocognitive assessment was done by the Neuropsychology Department, which highlighted the prominent deficits in orientation (space and time), working memory, episodic memory, as well as executive function (planning and monitoring, with a marked loss in error recognition).

Several antipsychotics and mood stabilizers were prescribed. When clozapine 200 mg and valproate 1200 mg were established, all symptoms had a good response except for the visual anosognosia and confabulations, which persisted.

## 3 Discussion

From a cognitive neuropsychiatric perspective, the classical triad of Anton syndrome characterizes the present case: blindness, anosognosia and confabulation [5]. Also, the patient showed the nihilistic delusions that define the presence of Cotard syndrome [6,7].

Anton described absence of the recognition of blindness (1896) in patients suffering from occipital lesions, [1,2] although there is historical evidence of previous observations in ancient Rome, by Seneca, the famous roman writer [8]. According to Babinsky (1914), anosognosia (a, without; noso, disease; gnosis, knowledge) is the loss of awareness of a neurological deficit, as seen in the hemiplegic subjects he studied. Confabulations, on the other hand, have been described by Berrios (1988) as an inaccurate or false narrative purporting to convey information about the world or self. The term was historically studied in patients with memory deficits caused by different pathologies, in which patients "cover" their deficit (untrue confabulations). Confabulations have also been described in the absence of amnesia, as in frontal lobe damage and in other psychiatric entities (fantastic confabulations) [9].

Although some subjects with cortical blindness present anosognosia and confabulations, most patients with cortical blindness do not, suggesting that bilateral occipital damage is not sufficient for the onset of anosognosia. The brain mechanisms behind visual anosognosia have not been explained [10]. Geschwind postulated that right hemisphere injuries could turn off right sensory monitors from the left frontal hemisphere, which tends to verbally deny deficits [11]. However, this theory does not account for the ability to express their deficits by means of non-verbal behavior [12]. This question was addressed by verifying that subjects with anosognosia for hemiplegia chose to tie their shoelaces with both hands instead of one [13]. This led to the proposal of a feed forward system where a sensory comparator center does not detect the dissociation between action and intention; therefore, the patient does not recognize the disability [14]. Recently, in vivo imaging studies suggested that this comparator might be located in the right lower parietal lobe [15].

Anosognosia and confabulations are useful in studying and locating brain dysfunction, particularly in the early stages of degenerative disorders, which modern neuroimaging techniques are still unable to detect. These naturalistic cases make us understand that clinical semiology is a useful tool to detect and address these exquisite phenomena. Also, lack of awareness of the disease by the patient can predispose to risky behaviors and poor family dynamics, which require an interdisciplinary approach.

Unlike the findings of motor anosognosia and the pathophysiology of confabulation, visual anosognosia has not been fully elucidated. However, there are some theories: Anton suggested that frontal areas of speech and language would be disconnected from damaged visual pathways, which starts the patient's confabulations to fill in the missing sensory information [1].

Anosognosia can be understood as a metacognitive deficit. Flavell defines metacognition as the subject's explicit knowledge about his or her own cognitive functioning [16]. Nelson and Narens (1990) proposed that metacognition emerges through a monitoring process that regulates the interaction with objects through a control process. This monitoring process is necessary to form a "metacognitive knowledge," and the control process generates a "metacognitive experience." These views have been shared by Flavell [16].

Unfortunately, as a limitation we should emphasize that due to the prominent visual deficit and the behavioral features of agitation, impulsivity, and lack of cooperation from the patient, the Neuropsychology Department could only make a qualitative assessment based on clinical interaction and bedside examination, without formal tests.

Our patient, JC, developed very specific cognitive deficits; visual anosognosia and confabulations after aneurysm clipping and during the subacute stage. As was mentioned, the pathophysiology of visual anosognosia has not been clearly established, probably because of the limited number of case studies. Nevertheless, visual anosognosia is possibly related to a monitor system as in motor anosognosia. Extensive or strategic damage to primary visual areas is probably a first neuropsychological process related to Anton syndrome, but our hypothesis is that it is not enough to develop the persistent anosognosia and confabulations observed in JC. The present case poses the requirement of a second neuropsychological factor: in this case, the damage in the frontoparietal cortex could provide the neuroanatomical substrate for a prolonged metacognitive deficit. Also, this was probably a factor involved in the formation of nihilistic delusions. As has been reported previously, nihilistic delusions have been studied within the concept of Cotard syndrome. This syndrome, involves frequently the delusion of “being dead” (as was expressed by our patient), and it has been associated with the right hemisphere lobe lesions or metabolic abnormalities in molecular imaging studies. [17], [18], [19], [20] The common mechanism of disturbed metacognition related to right fronto-parietal dysfunction contributes to explain why JC had both neuropsychiatric disturbances, Anton and Cotard syndromes.

This research did not receive any specific grant from funding agencies in the public, commercial, or not-for-profit sector.

## Declaration of Competing Interest

None.
